# Sneezes and Stitches

**Published:** 1858-06

**Authors:** 


### SNEEZES AND STITCHES.

IT may be well to know, that the very considerable inconvenience, *a stitch in the side*, is often removed in the same manner that a sneeze is prevented, to wit: by expiring all the breath possible from the lungs; push out the breath for half a minute or more, and draw it in slowly.

